# C-arm cone beam computed tomography with needle path overlay during fluoroscopic-guided screw placement for a spinal lesion

**DOI:** 10.1186/s12893-026-03709-2

**Published:** 2026-04-02

**Authors:** Hiroki Ohashi, Kohei Morita, Daichi Kawamura, Keisuke Hatano, Satoshi Yamana, Yuichi Murayama

**Affiliations:** Department of Neurosurgery, Jikei University School of Medicine, Tokyo, Japan

**Keywords:** Fracture Fixation, Spinal Fusion, Cone-Beam Computed Tomography, Fluoroscopy, Computer-Assisted

## Abstract

**Purpose:**

Accurate screw placement is essential in spinal surgery. To enhance precision, navigation-guided techniques using fluoroscopy or computed tomography (CT) have been developed. Recently, overlay technology has emerged, enabling real-time projection of preoperative planning onto fluoroscopic images through C-arm–based cone-beam CT (CBCT). Needle-guidance–based overlay technology was applied to the surgical management of odontoid fractures and pedicle screw placement, with accuracy and clinical outcomes systematically evaluated.

**Methods:**

A retrospective review was performed of three cases in which intraoperative overlay guidance using C-arm CBCT was considered effective for spinal surgery between February and June 2017. Case 1 involved a 26-year-old man with an Anderson type II odontoid fracture. Case 2 involved a 75-year-old woman with atlantoaxial dislocation. Case 3 involved a 66-year-old man with a vertebral arch fracture. For each case, postoperative imaging was used to assess the deviation between the planned trajectory and the actual implant position.

**Results:**

In Case 1, the deviation from the planned target point measured 2.57 mm. In Case 2, deviation were 0.71 mm (right C1), 0.98 mm (left C1), 0.35 mm (right C2), and 0.31 mm (left C2), producing a mean deviation of 0.59 mm. In Case 3, deviations were 0.88 mm (right L3), 2.21 mm (left L3), 2.37 mm (right L5), and 1.85 mm (left L5), resulting in a mean deviation of 1.83 mm. No major complications were observed in any of the three cases.

**Conclusion:**

Needle-guidance overlay technology extends beyond biopsy to encompass odontoid screw fixation and pedicle screw placement, demonstrating technical feasibility for accurate screw placement. In this study, implementation of the technique appeared to reduce the need for a separate navigation system without adding distinct invasive steps. It was also relatively straightforward to implement, suggesting feasibility even in smaller medical facilities.

## Introduction

Screw placement in the spinal region is vital when treating trauma or performing fusion surgeries for degenerative diseases. However, there is always a risk of serious complications, such as damage to nerve roots and blood vessels, owing to deviations during screw placement or incorrect screw placement. Since the 1980 s, efforts have been made to improve surgical accuracy using fluoroscopy and navigation techniques [1]. Since the first report of trauma treatments by Borne et al., fluoroscopy has been widely used to safely perform surgeries for odontoid process fractures [2]. Similarly, regarding the placement of pedicle screws, since first reported by Odgers et al., the surgery performed using fluoroscopy has replaced freehand placement owing to its high level of safety [3]. Since the development of computed tomography (CT), in addition to conventional fluoroscopy devices, it has been performed during surgery and used for surgical support. Haberland et al. demonstrated an improved navigation technology by using CT images captured during spinal surgery [4].

In particular, CT images are very useful because of their high image quality, but owing to space and cost restrictions, inclusion of CT equipment in an operating room has proven difficult [5]; thus, CT image acquisition using a C-arm has become popular in orthopedic surgeries. Kotani et al. reported cases of intraoperative navigation using an O-arm and placement of screws for safe surgery [5]. In the 2000 s, Hott et al. performed spinal surgeries using C-arm navigation. Since first reported, CT imaging and navigation devices have gained importance in spine surgery [6]. C-arm is widely utilized for fluoroscopy, and has greatly contributed to the advancement of minimally invasive surgeries. Since the 2,000 s, minimally invasive surgery has been widely performed using C-arm’s cone beam CT (CBCT) to avoid damage to the odontoid process and placement of pedicle screws, for intraoperative planning, and for setting and utilizing needle guidance [7, 8].

**Fig. 1:**
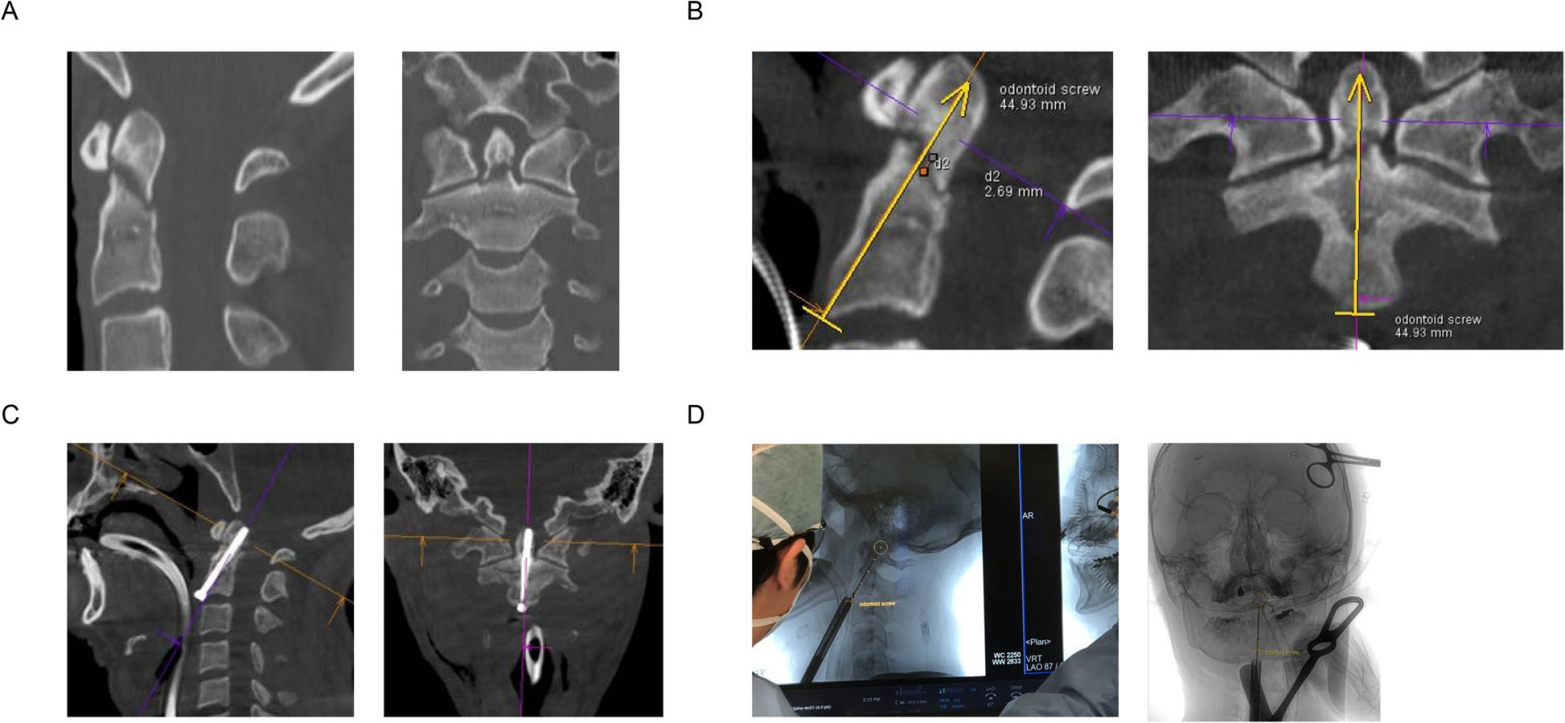
Overlay technology process (case 1). **A** Pre-operation C-arm cone beam computed tomography (CT) images. **B** Planning and determination of needle guidance. **C** Post-operation C-arm cone beam CT images. **D** Overlay image during operation

Under these conditions, a technology that reflects planning in fluoroscopy in real-time using CT images from C-arm has also emerged, and many reports have made effective use of needle guidance [8, 9]. This technology provides intraoperative support by planning trajectory of screws based on intraoperative images captured using C-arm during surgery on a workstation and reflecting them on a real-time fluoroscopic image (Fig. 1). Needle guidance is widely used, particularly in biopsy and drug infusion, because it allows for easy and safe linear entry [10, 11]. There are reports on vertebroplasty that used this method not only biopsy but also to perform advanced surgical techniques [12–16]. However, no reports of its use in the treatment of odontoid process fractures and pedicle screw placement. In this study, we planned to apply needle guidance to safely perform surgery for the treatment of odontoid process fractures and pedicle screw placement, and analyzed the results.

## Methods

### Patient demographics

We utilized an intraoperative support system using overlay technology for three surgical cases admitted to our institution in which planning by needle guidance was effective (Table 1). Freehand screw placement and fixation are associated with a high risk of complications. We selected cases that required safe and reliable device operation with linear planning. Particularly for case 3, accurate operation under fluoroscopy was essential, and linear planning accuracy was required to avoid complications; thus, overlay technology was the most feasible option. Informed consent was obtained from each patient, and the Ethical Commission of our university approved this study (# 31–060[9559]).

**Table 1:** Patient cases

Patient No	Sex	Age	Diagnos	Level	Operation style
1	Man	26	Odontoid fracture	C2	Odontoid process screw
2	Woman	75	Atlantoaxial dislocation	C1	Posterior fixation (C1-2)
3	Man	66	Verbal arch fracture	L4	Posterior fixation (L3-L5)

### Procedural technique and overlay technology

All procedures were performed by one or two neurosurgeons. The surgeries were performed under general anesthesia. Other preoperative, intraoperative, and postoperative systemic management procedures were performed using the same procedure, and there was no difference in the procedure using the overlay technology. Overlay technology describes the use of a three-dimensional (3D) image dataset to plan the placement of screws. Afterwards, each live fluoroscopic image shows the actual trajectory of a planned 3D pathway on a two-dimensional (2D) image.

Immediately before or during the operation, CBCT was performed using a robotic Angiography System ARTIS pheno C-arm (Siemens Healthcare, Erlangen, Germany), which offers CT-like imaging with a volume of 23 × 17 cm (height × diameter) at a resolution of 512 × 512 pixels per slice, and a voxel size of 0.33 mm. The system was also used as an intraoperative imaging system, and the fluoroscopy CBCT images were sent to a workstation (ARTIS pheno, VE 10 B, Siemens Healthcare GmbH, Erlangen, Germany). In the workstation, a model was three-dimensionally reconstructed using a liquid–crystal display with a resolution of 1,920 × 1,200 dots (EIZO Corporation, Hakusan, Japan), and intraoperative planning was conducted based on the model. The needle guidance pathway created based on the 3D model was displayed as an overlay on fluoroscopic display and a liquid–crystal display with a resolution of 1,920 × 1,200 dots (Siemens Healthcare GmbH, Erlangen, Germany). In summary, the system captures CBCT images immediately before the start of a surgery after a patient is positioned, converts these images into a 3D model in the workstation, creates a pathway plan on the spot, and reflects it in intraoperative fluoroscopic images. Therefore, there is no need for continuous sensing during surgery, and CBCT images are not required to be retaken during surgery. We used an intraoperative support system with overlay technology (Fig. 1). In each case, the magnitude of the difference between a planned placement schedule and an actual placement in a postoperative image, and the presence or absence of complications were measured and analyzed.

The planned placement position was determined from data obtained during preoperative planning, while the actual placement position was assessed using postoperative CT images. Both evaluations were performed with the same workstation software employed for planning, and the error was identified. Error assessment was consistently conducted by a single neurosurgeon.

The deviation between the planned and actual screw positions was calculated as the three-dimensional Euclidean distance between the planned target point and the actual screw tip on postoperative CT. The workstation automatically aligned preoperative planning data and postoperative images using rigid registration based on vertebral bony landmarks. The same anatomical reference point—the screw tip—was used for all measurements. Because all measurements were performed by a single neurosurgeon and no interobserver comparison was performed, the absence of interobserver validation represents a methodological limitation of this study.

## Results

### Case 1

A 26-year-old man presented with neck pain following a traffic accident. A CT showed an Anderson type 2 odontoid fracture, which required fixation after unsuccessful immobilization. During screw placement (diameter, 3.5 mm; length, 42 mm), the drill approached the deepest point of the screw. This screw target point was calculated using a needle guidance software and the approach described above. After the surgery, CBCT confirmed that the screw was placed in a safe area as planned (Fig. 1). Target point deviation was 2.57 mm.

### Case 2

**Fig. 2:**
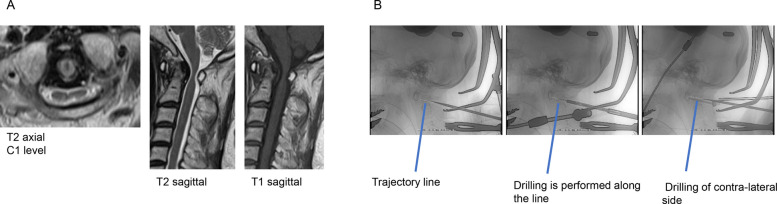
Presentation of case 2. **A** Pre-operation magnetic resonance imaging images. **B** Insertion of screws along the virtual insertion line

A 75-year-old woman presented with bilateral dysesthesia and mild myelopathy; she subsequently underwent surgery. The patient had prominent spinal cord compression at C1 level on preoperative magnetic resonance imaging (MRI); therefore, posterior fixation (C1–2) was required. During intraoperative planning, trajectory of the screws was determined, overlaid, and displayed on a fluoroscopic image under needle guidance. We drilled along an ideal trajectory line and placed the screws (C1 right side: diameter 3.5 mm and length 28 mm; C1 left side: diameter 3.5 mm and length 28 mm; C2 right side: diameter 3.5 mm and length 20 mm; and C2 left side: diameter 3.5 mm and length 30 mm). All the surgical procedures were performed under fluoroscopy. Postoperatively, CBCT confirmed that the screws were placed in safe areas, as planned (Fig. 2). Target point deviation was 0.71 mm on the right side of C1, 0.98 mm on the left side of C1, 0.35 mm on the right side of C2, and 0.31 mm on the left side of C2. Average target point deviation was 0.59 mm.

### Case 3

**Fig. 3:**
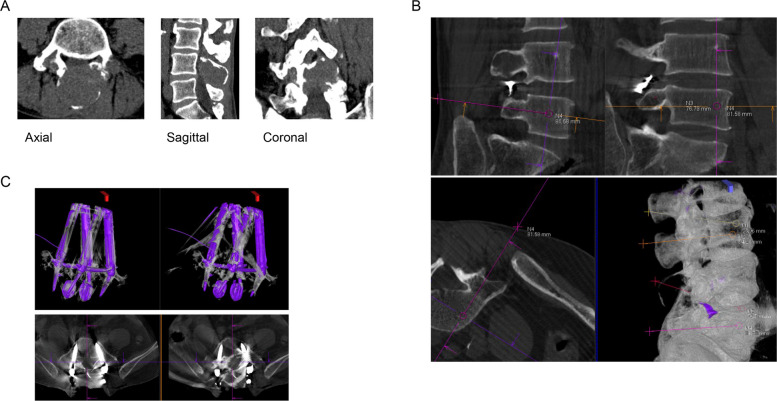
Presentation of case 3. **A** Pre-operation computed tomography (CT) images. **B** Planning determination of needle guidance on workstation. C. Post-operation CT and three-dimensional images

A 66-year-old man who underwent total nephrectomy for left renal cell carcinoma 4 years prior to admission was followed up. A neoplastic lesion appeared in the L4 pedicle at 1 year prior to admission. He was administered chemotherapy with sunitinib because of the gradual increase; however, because of severe symptoms, such as loss of appetite, the treatment was discontinued, and radical removal surgery was planned. No physical findings, including myelopathy or radiculopathy, were observed on admission. However, CT imaging showed that the L4 vertebral arch had osteolysis due to a neoplastic lesion, and the spinal canal was slightly compressed from the dorsal side (Fig. 3A). Embolization before surgery was planned because angiography showed significant arterial supply to the tumor from the left and right L4 lumbar arteries. Therefore, embolization using microspheres and coils was performed on the right side, while arterial embolization was performed on the left side using coil embolization alone. Next, L4 tumor removal and L3–5 posterior fusion were performed. A posterior approach was used, and after total removal of the L4 vertebral arch tumor, the L3/4 and L4/5 discs were removed, followed by posterior fusion. During intraoperative planning, location of the screw placement in each vertebral body was set by entering the entry and endpoints of the desired needle path into the acquired 3D image (Fig. 3B). An overlay was created on fluoroscopic images for needle guidance. During placement of the screws, their position was continuously confirmed under fluoroscopy. The screws used were all 6.5 mm in diameter and 40 mm in length, at the right and left sides of L3 and right and left sides of L5. After the surgery, CBCT confirmed that the screws were placed in safe areas as planned (Fig. 3C). Target point deviation was 0.88 mm on the right side of L3, 2.21 mm on the left side of L3, 2.37 mm on the right side of L5, and 1.85 mm on the left side of L5. Total average deviation was 1.83 mm.

In all the three cases, there were no major complications such as blood vessel or nerve damage due to screw deviation, and no complications clearly attributable to the procedure occurred.

## Discussion

Overlay technology reflects planning in fluoroscopy in real time using CT images from the C-arm. Since 2010, there have been multiple reports using needle guidance. Alda et al. first reported that needle guidance could be performed safely and effectively under fluoroscopy using an overlay technology for vertebroplasty [8]. With linear planning and approach route settings, biopsy and drug injection were described by Alda et al. as safe and reliable. Therefore, needle guidance is considered effective for various interventions and surgical procedures. Because many of these procedures are minimally invasive, ensuring safety is of utmost importance for better patient outcomes. Greater improvements in image quality are the reasons why fluoroscopy is often used in minimally invasive surgeries. Additional overlay technologies have resulted in safer and more reliable surgical procedures. Additionally, needle guidance is widely used, particularly in biopsies; this is because it allows easy and safe straight-line entry, which is highly effective [10, 11]. Regarding the application of this system not only for biopsy but also for advanced surgical techniques, reports have shown that it is effective for vertebroplasty, as described above, and there are also reports of its efficacy in catheter insertion [8, 17]. However, there are few reports on the use of devices other than needles [18, 19], and there are no reports on their active use in odontoid process surgery or pedicle screw placement.

Recently, the use of intraoperative navigation systems in procedures with odontoid process fracture repair or pedicle screw placement has become common. In intraoperative navigation, it is common to perform linear planning using needle guidance to avoid complications and safely perform a surgery. Cases involving navigation systems with intraoperative C-arm images have been reported since 2000 by Langston et al. among many others [7]. Additionally, for repair of odontoid process fractures, Lori et al. reported that surgery could be effectively performed under a navigation system using needle guidance [9]. Thus, linear planning is important for odontoid process fracture repair and pedicle screw placement, and it is compatible and can be performed effectively with needle guidance. We performed needle guidance using the overlay technology and reported its technical feasibility in these cases. In case 1, the fractured and released odontoid process moved easily, and the navigation could not display the released bone fragments directly; therefore, operation under fluoroscopy was essential, and needle guidance was effective for safe and accurate screw placement.

In the three cases, the target deviation was between 0.59–2.57 mm, as described above. For context, reported mean deviation values for fluoroscopy-assisted pedicle screw placement typically range from 1.0 to 3.5 mm, while navigation-assisted techniques (O-arm, CT-based) generally achieve mean deviations of 0.5 to 2.0 mm [20, 21]. The deviations in our three cases were small, and most of the errors observed were thought to be as a result of the device insertion procedure, rather than errors caused by the system. Potential sources of errors outside the system itself include instrument rigidity, bone displacement, and surgical technique. Among these, instruments are composed of metal and are highly resistant to abnormal deformation during insertion; indeed, such stress would be hazardous, so their contribution to error is negligible. Furthermore, when the surgical procedure is performed within the scope of safe and reliable methods, techniques that generate substantial errors are dangerous, and their contribution to overall error is minimal. In contrast, unavoidable bone displacement associated with the procedure—such as shifts caused by instrument pressure—is likely to contribute substantially to error. The target deviation values observed ​​in this study are logically consistent with the distances attributable to bone displacement during the procedure. Therefore, errors arising from unavoidable bone displacement constitute the predominant source of deviation. In light of this, the pursuit of safe and accurate screw placement remains imperative, as emphasized above.

This study did not compare instruments with other existing systems as a control group. Therefore, it does not demonstrate superiority through quantitative analysis. However, the advantages and characteristics of a system can be characterized not only by error metrics but also by factors such as actual clinical applicability and implementation costs. Accordingly, the presence or absence of clinical complications, together with the outcomes, holds significance in the present study. As a clinical problem, it is extremely unlikely to cause protrusion into the spinal canal if it deviates from the center line of the odontoid process. Therefore, the risk of adverse events is low. In fact, in all the three cases, there were no complications related to the overlay technology, including mild ones, and it is considered that there was no problem with safe operations.

Our method uses overlay technology with C-arms in combination with its workstations rather than an additional dedicated navigation system. First, when using a normal navigation system for surgery, it is necessary to fix a registration point to the spinous process to perform registration, but this is unnecessary in our method, and it does not require an additional device in the surgical field. Therefore, no additional skin incisions for registration, including skin incisions, are required to reduce the burden on patients. Because it is displayed in real time, there is little difference with the previous registry. The surgical field and development can simply be summarized, which may reduce the burden on surgeons. Second, a system that uses C-arm is cheaper than introducing a new navigation system as a device to guide and support surgery. This is an important characteristic of this system, as it makes it easy to guide and support surgery, even in medical institutions that are not large enough to introduce navigation. Surgical navigation systems utilize O-arm, C-arm, and conventional CT images during surgery. Each of these methods has its own advantages and disadvantages, and it is important to choose the appropriate method, depending on the circumstances of the doctor and hospital [20, 21]. Nine years have passed since the development and use of this system began, but the system itself has not changed, the method of use is stable, and our hospital continues to use it as a cost-effective method.

**Fig. 4:**
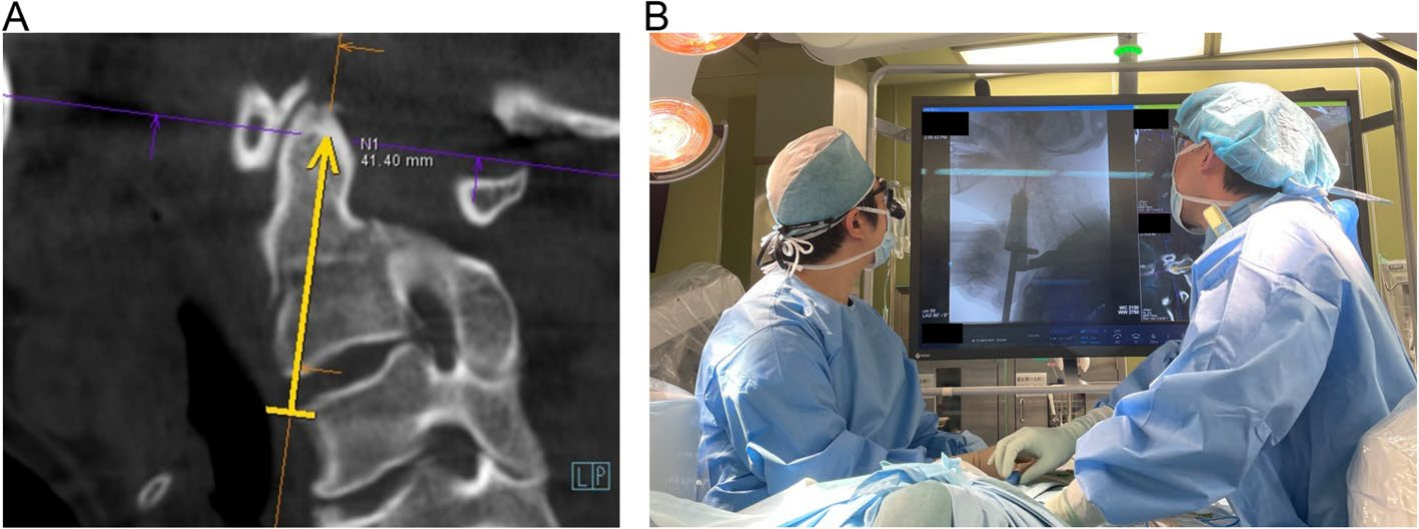
Guidance of young doctors by an experienced doctor. **A** Ideal procedure planned by an experienced doctor (odontoid process screw insertion). **B** Guidance of intraoperative procedures for young doctors using overlay technology

Additionally, reduced treatment burden can be advantageous for educational medical institutions and for the guidance of students and young doctors. Medical education and guidance can be instigated by the efficient study of surgical planning by experienced instructors. As mentioned above, subjective planning by a surgeon is the most important factor in avoiding complications because an error due to intraoperative planning and surgical operation is superior to that due to the overlay technology itself. In particular, with regard to the guidance of young doctors, it is ideal to perform a procedure planned by an experienced doctor (Fig. 4A) and correct errors that may occur during surgery (Fig. 4B). Possible sources of error include misalignment of the hand, bending of the needle, and improper penetration of the needle during insertion, which causes the target bone to move. Although it is extremely difficult to completely avoid these complications, it is clear that they are more likely to occur if the surgical techniques are performed in an unreasonable manner or with an excessive load. The chance of avoiding errors is increased by adhering to the basics of surgical techniques and not performing unreasonable operations. Furthermore, when a misalignment is clearly large, it is generally best to continue the procedure while relying on fluoroscopic images.

This study has limitations. Although the models on CT and workstations were anatomically consistent, there may be case-to-case differences in terms of technical points, operator preferences, radiation exposure dose, and visibility of CT and fluoroscopy. In particular, a quantitative comparative assessment of radiation exposure has not been performed. Published data on CBCT-based spinal workflows report operator doses in the range of 0.01–0.1 mSv per procedure, comparable to conventional fluoroscopy-guided approaches [20]. Because the same system as conventional fluoroscopy is employed in our technique, the exposure mechanism is identical and proportional to the duration of the surgical procedure. The use of this system may facilitate smoother operative workflows, which could shorten operative time and thereby reduce radiation exposure compared with existing techniques. Nonetheless, a formal quantitative evaluation has not yet been conducted. The technical limitation is that the patient must remain in the same position on the operating table throughout the procedure, which can lead to errors in the position and orientation of the target point, depending on movement during the procedure. This problem is similar to that in the conventional navigation system; thus, continuous advancements in the technologies used are required to further improve accuracy. Depending on the case and surgical operation, exposure dose during fluoroscopy increases, which is a disadvantage compared with general navigation use. Lastly, there was difficulty in evaluating the magnitude of the errors and deviations in this system. Ideally, we should compare its accuracy with that of the conventional navigation systems, and the effect of deviations on surgical techniques. However, the differences between this system and the conventional navigation systems, such as image evaluation timing and the presence or absence of sensing, make direct comparisons difficult. Furthermore, as mentioned above, deviations can occur because of the surgical techniques, making it nearly impossible to evaluate their superiority or inferiority. Therefore, in this study, we limited the quantitative evaluation to the numerical values ​​of the final results and proposed a method for evaluating the usefulness of this technique.

## Conclusion

Overlay technology, such as needle guidance, which reflects planning of a screw pathway on a 2D fluoroscopic image using ARTIS pheno, a fixed robotic C-arm, can be applied not only to biopsy but also to placement of the odontoid process and pedicle screw, suggesting technical feasibility for these applications. This approach does not require an independent navigation system and appears straightforward to implement, even in small-scale facilities. Overlay technology is expected to become more widely explored for intraoperative support systems. As this is only a small case series from a single center, additional prospective comparative studies with larger patient cohorts and a multicenter approach are needed to formally evaluate accuracy, safety, and efficiency relative to established techniques.

## Data Availability

This study was not registered in any public database. The data were kept strictly confidential by the authors and can be provided upon request. Contact information Kohei Morita, M.D. PH.D. [E-mail: kmorita710@gmail.com], Jikei University School of Medicine, Nishi-Shinbashi 3–25-8, Minato-ku, Tokyo, 105–8461, Japan TEL: (+ 81) 3–3433-1111 Fax: (+ 81) 3–3459-6412.

## Abbreviations

CT: Computed Tomography
CBCT: Cone beam CT
3D: Three-dimensional
2D: Two-dimensional
MRI: Magnetic resonance imaging
